# Detection and Investigation of Eagle Effect Resistance to Vancomycin in *Clostridium difficile* With an ATP-Bioluminescence Assay

**DOI:** 10.3389/fmicb.2018.01420

**Published:** 2018-07-02

**Authors:** Angie M. Jarrad, Mark A. T. Blaskovich, Anggia Prasetyoputri, Tomislav Karoli, Karl A. Hansford, Matthew A. Cooper

**Affiliations:** Institute for Molecular Bioscience, The University of Queensland, St. Lucia, QLD, Australia

**Keywords:** *Clostridium difficile*, Eagle effect, vancomycin, antibiotic resistance, ATP-bioluminescence

## Abstract

Vancomycin was bactericidal against *Clostridium difficile* at eightfold the minimum inhibitory concentration (MIC) using a traditional minimum bactericidal concentration (MBC) assay. However, at higher concentrations up to 64 × MIC, vancomycin displayed a paradoxical “more-drug-kills-less” Eagle effect against *C. difficile*. To overcome challenges associated with performing the labor-intensive agar-based MBC method under anaerobic growth conditions, we investigated an alternative more convenient ATP-bioluminescence assay to assess the Eagle effect in *C. difficile*. The commercial BacTiter-Glo^TM^ assay is a homogenous method to determine bacterial viability based on quantification of bacterial ATP as a marker for metabolic activity. The ATP-bioluminescence assay was advantageous over the traditional MBC-type assay in detecting the Eagle effect because it reduced assay time and was simple to perform; measurement of viability could be performed in less than 10 min outside of the anaerobic chamber. Using this method, we found *C. difficile* survived clinically relevant, high concentrations of vancomycin (up to 2048 μg/mL). In contrast, *C. difficile* did not survive high concentrations of metronidazole or fidaxomicin. The Eagle effect was also detected for telavancin, but not for teicoplanin, dalbavancin, oritavancin, or ramoplanin. All four pathogenic strains of *C. difficile* tested consistently displayed Eagle effect resistance to vancomycin, but not metronidazole or fidaxomicin. These results suggest that Eagle effect resistance to vancomycin in *C. difficile* could be more prevalent than previously appreciated, with potential clinical implications. The ATP-Bioluminescence assay can thus be used as an alternative to the agar-based MBC assay to characterize the Eagle effect against a variety of antibiotics, at a wide-range of concentrations, with much greater throughput. This may facilitate improved understanding of Eagle effect resistance and promote further research to understand potential clinical relevance.

## Introduction

*Clostridium difficile* is a spore forming, anaerobic bacteria that causes colon inflammation and diarrhea, which in severe cases can be life threatening. Over the last 15 years, *C. difficile* has become a significant threat to human health and a billion dollar economic burden (Centers for Disease Control and Prevention, 2013). *C. difficile* infections (CDIs) are usually treated orally with metronidazole, vancomycin or fidaxomicin antibiotics; in recent years fecal transplant has become an important alternative investigational biotherapeutic to treat severe recurrent disease (Jarrad et al., 2015). Metronidazole is only recommended to treat mild to moderate cases, while vancomycin and fidaxomicin have improved cure rates relative to metronidazole for more severe infections (Crook et al., 2012; Surawicz et al., 2013). Metronidazole is nearly completely systemically absorbed following oral dosing, resulting in relatively low concentrations of antibiotic at the desired location of the site of infection in the colon (Bolton and Culshaw, 1986). In contrast, vancomycin and fidaxomicin have minimal systemic absorption, resulting in high concentrations in the colon (>1000 μg/g) (Sears et al., 2012). Fidaxomicin therapy is associated with reduced relapse rates compared to vancomycin and metronidazole. The capacity of fidaxomicin to inhibit spore formation (Babakhani et al., 2012) and the enhanced selectivity of fidaxomicin for *C. difficile* over beneficial gut microbiota compared to vancomycin (Louie et al., 2012) are thought to contribute to this outcome.

The bactericidal nature of antibiotics is an important property assessed during drug development. Fidaxomicin and metronidazole are bactericidal (Goldstein et al., 2010; Babakhani et al., 2011), as are investigational CDI candidate antibiotics surotomycin and cadazolid (Locher et al., 2014; Mascio et al., 2014), recently assessed in phase III clinical trials for treatment of CDI (Daley et al., 2017)^1^. In contrast, vancomycin has been reported to be bacteriostatic (Odenholt et al., 2007). The minimum bactericidal concentration (MBC) is the concentration of compound that results in a 99.9% reduction in the number of viable bacteria compared to the initial inoculum. One definition of a bactericidal compound is an MBC to minimum inhibitory concentration (MIC) ratio of 4 or less, although debate exists over the clinical relevance of bacteriostatic versus bactericidal mode of action (Pankey and Sabath, 2004; Wald-Dickler et al., 2017).

The MBC can be determined with different methods. In time-kill assays, bacterial viability (CFU/mL) is measured in the presence of varied concentrations of antimicrobial agent above the MIC over a 24 h time course. The MBC can also be determined at a single time point. After conducting a broth-based MIC assay, aliquots from wells with no visible growth are plated onto agar and the number of colonies that grow after 24 h is determined. The MBC is the concentration of compound where the number of viable colonies is less than a threshold value outlined in the NCCLS standard (NCCLS, 1999). This threshold value accounts for sampling error and the initial inoculum concentration and is equivalent to ≤0.1% surviving bacteria (NCCLS, 1999).

The Eagle effect, described by Harry Eagle in 1948, occurs when high concentrations of antibiotic above the MBC result in an increased proportion of surviving cells (Eagle, 1948). Bacterial cells exposed to higher concentrations of antibiotic exhibited a decreased rate of cell death in this study (Eagle, 1948). The Eagle effect has been reported for a range of microbial species [Gram positive, Gram negative, mycobacterium (Wu et al., 2015) and fungi (Fleischhacker et al., 2007)] and antibiotics [β-lactams (Eagle, 1948), fluoroquinolones (Wu et al., 2015), aminoglycosides (Lorian et al., 1979), glycopeptides (McKay et al., 2009), lipopeptides (Annear, 1970)]. Despite, the wide range of organisms and antibiotics displaying this paradoxical “more-kills-less” response, including in *in vivo* efficacy models (Ikeda et al., 1990; Grandière-Pérez et al., 2005), the molecular mechanism has not yet been determined for all agents. For cephalosporins, a type of β-lactam antibiotic, the paradoxical effect has been explained by high concentrations of antibiotic inducing increased levels of β-lactamase that inactivate the drug to a greater extent (Ikeda et al., 1990).

In the course of determining the MBC of compounds against *C. difficile* strain 630 (ATCC BAA-1382) by the traditional micro-broth dilution CFU-based method (NCCLS, 1999), we observed an Eagle effect for vancomycin, but not metronidazole. This Eagle effect for vancomycin-treated *C. difficile* has previously been observed, with literature reports of paradoxical, concentration independent killing by vancomycin against several strains of *C. difficile* in time-kill experiments (Levett, 1991; Odenholt et al., 2007; Corbett et al., 2015). Given that vancomycin reaches supra-MIC levels in the human colon during oral dosing for treatment of CDI, we wished to further investigate Eagle-type resistance in *C. difficile*. Unfortunately, CFU plating methodology is low throughput. After the broth micro-dilution MIC assay, aliquots from wells of a 96-well plate with inhibited bacterial growth (i.e., no turbidity) are spread plated onto respective agar plates. These plates are incubated for 24 h and the bacterial colonies counted. When conducted in an anaerobic chamber as required for *C. difficile* growth, the aliquot and spread plate steps of the procedure are cumbersome. The agar-based MBC assay therefore has limited scalability, making it difficult to investigate more than one antibiotic at a wide range of concentrations and assess activity against different strains of bacteria. In addition, the MBC assay has another limitation, that antibiotic carry-over from the MIC well onto the agar plate can affect the subsequent growth of bacteria into colonies (NCCLS, 1999). We therefore investigated the use of a commercial ATP-bioluminescence assay as an alternative, operationally simple method with greater throughput to detect, and facilitate our study of the Eagle effect in *C. difficile*.

The commercial ATP-bioluminescence assay (BacTiter-Glo^TM^) uses a proprietary thermostable luciferase in combination with a formulation that extracts ATP from metabolically active cells. A luciferase catalyzed reaction of Beetle Luciferin and ATP generates a stable glow-type luminescent signal which is an indirect measure of bacterial cell viability (Promega Corporation, 2012). This end-point detection method is independent of further bacterial growth and is operationally simple and quick due to the add – mix – measure format. Reagent addition, incubation and measurement is performed in an aerobic environment to provide molecular oxygen necessary for the ATP-bioluminescence reaction. Therefore, cumbersome manipulations associated with maintaining an anaerobic atmosphere necessary for *C. difficile* growth are reduced compared to CFU plating methodology. We report here investigation of the Eagle effect in *C. difficile* with ATP-bioluminescence for clinically relevant concentrations of current CDI antibiotic treatments, for a range of different lipoglycopeptides and against multiple pathogenic strains of *C. difficile* strains.

## Materials and Methods

### Antimicrobial Compounds

Vancomycin, metronidazole, and ramoplanin were commercially available from Sigma-Aldrich. Oritavancin was commercially available from MedKoo Biosciences, Inc. Fidaxomicin was obtained from Optimer via Specialised Therapeutics Australia in tablet form and the compound for assay was obtained by extraction with methanol and subsequent evaporation of volatiles *in vacuo*. Dalbavancin and telavancin were synthesized according to literature procedures with the structures supported by NMR, HRMS, and MS-MS analysis (Leadbetter et al., 2004; Cheng et al., 2014b). Purity of fidaxomicin and the synthesized compounds was ≥95% as determined by LC-(+)-ESI-MS/UV/ELSD. Compound stocks (20×) were prepared in water, and diluted in media for the assay. For testing metronidazole at 2048 μg/mL, the compound was dissolved directly in assay media at 2× final concentration. Fidaxomicin stock was prepared in DMSO (104.2 mg/mL) and diluted with media to final assay concentrations of DMSO ≤0.3% for assays up to 4 μg/mL and to ≤2% for testing up to 2048 μg/mL.

### Minimum Inhibitory Concentration

Minimum inhibitory concentrations against *C. difficile* strains (630 = ATCC BAA-1382; two “hypervirulent” NAP1/027 = ATCC BAA-1803 and M7404; high-level toxin producer VPI10463 = ATCC 43255) were determined as previously described (Jarrad et al., 2016). Briefly, 20× antibiotic stock solutions were diluted 1:10 in media and serially diluted twofold in 96-well plates (Non-binding surface, Corning Inc., Corning, NY, United States). The plates were placed in a Coy anaerobic chamber (5% H_2_, 10% CO_2_, 85% N_2_) overnight to reduce the medium. *C. difficile* bacteria from BHIS(TA) (Brain heart infusion-supplemented plus 0.1% taurocholate) agar plates were cultured anaerobically in BHIS at 37°C overnight. A sample of culture was then diluted 40-fold in BHIS broth and incubated at 37°C for approximately 4.5 h. The resultant mid-log phase culture (OD_600_ = 0.4–0.6) was diluted to a final concentration of ∼1 × 10^6^ CFU/mL, then 50 μL was added to each well of the compound-containing 96-well plates, yielding a final cell concentration of 5 × 10^5^ CFU/mL and final volume of 100 μL. Final compound concentration in each row ranged from 2048 to 1, 64 to 0.03 or 4 to 0.002 μg/mL. An antibiotic standard, positive growth control (no compound, water or DMSO vehicle) and sterility control (no bacteria) rows were included on each 96-well plate. Plates were covered and incubated at 37°C for 24 h. MICs for each strain were determined as the lowest concentration without visible growth. MICs were determined in duplicate with at least two independent experiments. Variance between replicates was typically within one twofold dilution. Median MICs are reported with a range given when the median MIC was between two tested concentrations.

### Minimum Bactericidal Concentration

Minimum bactericidal concentrations were determined according to NCCLS Methods for Determining Bactericidal Activity of Antimicrobial Agents M26-A (NCCLS, 1999). Briefly, after MIC determination, duplicate 10 μL volumes from each well of interest from one of the MIC replicate plates were spread onto pre-reduced BHIS agar plates inside of the anaerobic chamber. The well containing the lowest amount of compound with visible growth was plated as a control and the wells with increasing concentrations of compound at and above the MIC with no visible growth were plated. The plates were placed in a bag to reduce desiccation and incubated for 24 h. The numbers of colonies were counted and the MBC determined according to the reported threshold that corresponded to a 99.9% reduction in growth compared to the initial inoculum CFU/mL. The median MBC was reported. For metronidazole (*n* = 4, two independent duplicate experiments) all provided MBC = 0.5 μg/mL, while for vancomycin (*n* = 6, three independent duplicate experiments), one duplicate MBC was 4 μg/mL and the other two 8 μg/mL.

### ATP-Bioluminescence Assay

The ATP-Bioluminescence assay (BacTiter-Glo^TM^, Promega, Madison, WI, United States) was performed according to the manufacturer’s instructions (Promega Corporation, 2012). To maximize the sensitivity, BacTiter-Glo^TM^ Substrate and Buffer were mixed ∼2 h *prior* to measurement to allow for residual ATP “burn–off.” The broth micro-dilution MIC assay for *C. difficile* was performed in parallel to the MIC assay described above, except white assay plates (96 well, white NBS, Corning Inc., Corning, NY, United States) suitable for luminescence detection were used. After 24 h incubation, the assay plates were equilibrated to room temperature and then removed from the Coy anaerobic chamber (5% H_2_, 10% CO_2_, 85% N_2_). ATP-bioluminescent reagent was added in equal volume to the bacterial culture (100 μL of reagent to 100 μL of bacterial culture). The assay plates were briefly shaken (1 min, 300 RPM) to lyse the bacterial cells and were then equilibrated for 5 min in the dark. The total unfiltered relative luminescence units (RLU) were measured with a microplate reader (Tecan Infinite M1000 PRO), integration time = 1000 ms, and then normalized to the positive growth control (no antibiotic) according to the formula:

$$\begin{array}{c} \%Relative Luminescence =(well luminescence-mean of media background)/(mean of growth control-mean of media background)*100\%. \end{array}$$

We note that it is necessary to normalize the raw luminescence data because while the BacTiter-Glo is considered a stable luminescence (vs. flash based luminescence), the signal decays (half-life 30 min). Therefore, in order to compare results acquired from different plates and independent assays, the data was normalized to the mean of positive growth control wells included on each assay plate. The average background from the negative growth control wells (media only) on each assay plate was subtracted as detailed in the methods. Viability data in the manufacturer’s technical bulletin is normalized and presented as % RLUs.

To determine the assay quality the *Z’* score and signal to noise was calculated for each assay plate and the means reported ± SEM. Mean RLU of media background and positive growth controls are reported ± SEM. Minimum *n* = 6 ± SEM (at least three independent experiments). Data were processed in Microsoft Excel 2017 and analyzed in GraphPad Prism 7.

## Results

### Eagle Effect for Vancomycin in a Microbroth Dilution MBC Assay

The MBC of metronidazole, determined by broth microdilution against *C. difficile* strain 630, was equal to the MIC value of 0.5 μg/mL (*n* = 4). Vancomycin was bactericidal at 8 μg/mL, 8 × MIC (1 μg/mL), but with higher concentrations of antibiotic (16–64 × MIC) more bacteria survived (*n* = 6). At 8 × MIC of vancomycin, this equated to 5.5 × 10^2^ CFU/mL surviving cells. Accurate quantification of CFU from samples incubated with 32–64 × MIC vancomycin was difficult due to the density of bacterial growth on the agar. Nonetheless, at 64 × MIC there were approximately 1.25 × 10^4^ CFU/mL, an increase of ∼1.4 log_10_ surviving bacteria. This level of surviving bacteria was consistent with literature. For example, in time-kill experiments, *C. difficile* strain BI1 exposed to 32 × MIC vancomycin resulted in 1.2 log_10_ CFU/mL more surviving bacteria after 24 h than at 4 × MIC (Corbett et al., 2015). In addition, *C. difficile* 382 exposed to 20 × MIC had ∼1.6 log_10_ CFU/mL more surviving cells after 24 h than at 2 × MIC (Odenholt et al., 2007). We wish to note that, the reproducible nature of the Eagle effect in *C. difficile* in our hands differs from other bacterial strains, such as *Staphylococcus aureus*, where our attempts to replicate literature reports of the Eagle effect have been largely unsuccessful (data not shown).

### ATP-Bioluminescence Can Detect Eagle-Type Resistance in *C. difficile*

**FIGURE 1:**
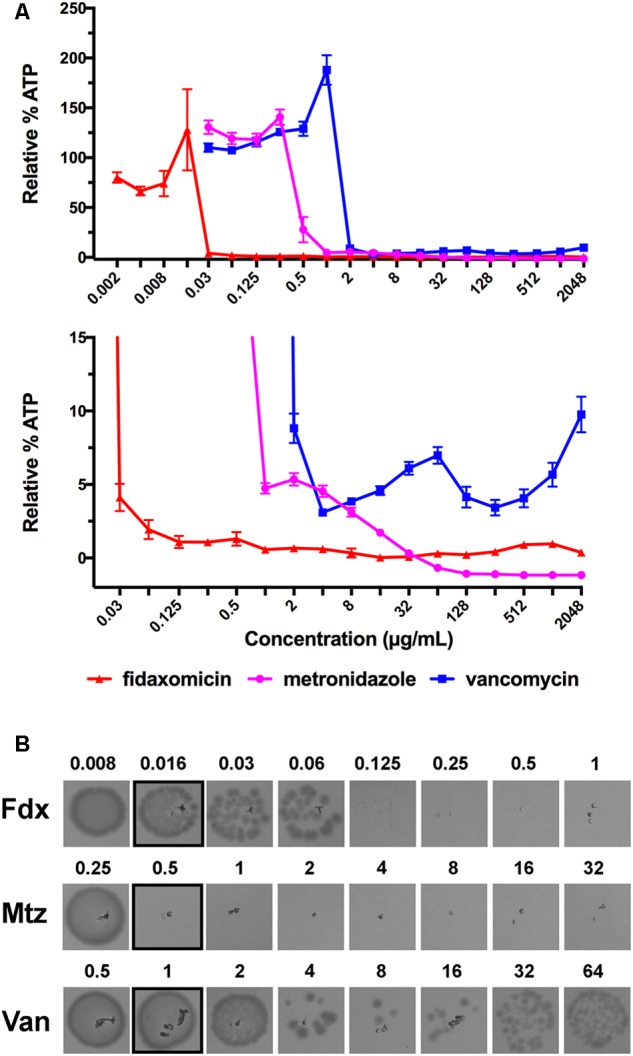
Detection of the Eagle effect for vancomycin, but not metronidazole or fidaxomicin, in *C. difficile* 630. **(A)** ATP-Bioluminescence after 24 h incubation of metronidazole, fidaxomicin, and vancomycin, and *C. difficile* (up to 2048 μg/mL of compound). Top panel: complete graph, bottom panel: expansion of top panel with % Relative Luminescence ≤15%. Minimum *n* = 6 ± SEM. **(B)** Growth on BHIS agar after 24 h incubation from aliquots (5 μL) of metronidazole, fidaxomicin, and vancomycin treated *C. difficile* (μg/mL). The concentration range was 0.5–64 × MIC, with the growth corresponding to the MIC highlighted with a black box. Representative image of *n* = 6.

We next measured the ATP-bioluminescence of *C. difficile* 630 after 24 h incubation with metronidazole, fidaxomicin, and vancomycin up to 2048 μg/mL (**Figure 1A**). The high concentrations tested approximate the concentrations achieved in the colon for fidaxomicin and vancomycin during clinical treatment. Metronidazole and vancomycin were soluble at all concentrations, while fidaxomicin was a suspension at the three highest concentrations tested (512–2048 μg/mL). In addition to luminescence assays, MICs were determined in parallel using clear 96-well plates (**Table 1**). Clear bottomed white wall 96-well plates are available which would permit MIC determination followed by ATP-luminescence measurement in the same plate. However, these were not used in this study because they can result in lower signal intensity and greater cross-talk between wells (Promega Corporation, 2012). The MIC for these three antibiotics corresponded closely to the concentrations at which large reductions in the % bioluminescence were observed.

**Table 1:** MIC of antibiotics against four strains of *C. difficile*.

	MIC (μg/mL)				Eagle effect
	630^a^	VPI10463^b^	ATCC BAA-1803	M7404	
Fidaxomicin	0.016	0.03	0.06	0.125	–
Metronidazole	0.5	0.5	0.5	0.5	–
Vancomycin	1	1	2	1	+
Telavancin	0.125	0.125	0.125	0.06	+
Teicoplanin	0.125	0.25	0.25	0.125	–
Dalbavancin	0.03	0.03	0.06	0.016	–
Oritavancin	0.125	N.D.	N.D.	0.06	–
Ramoplanin	0.03	0.06	0.03	0.03	–

*The MICs were determined in duplicate with at least 2 independent experiments performed for each compound. (+) = Eagle effect, (-) No Eagle effect. ^*a*^ATCC BAA-1382. ^*b*^ATCC 43255.*

To validate that the increase in luminescence at concentrations of vancomycin >4 μg/mL corresponded to an increase in viable cells, duplicate samples of culture (5 μL) from a replicate assay MIC plate were spotted onto BHIS agar and incubated for a further 24 h under anaerobic conditions (**Figure 1B**). Samples were also tested on BHIS agar supplemented with 0.1% sodium taurocholate germinant; no growth difference was observed between the two agars for metronidazole or fidaxomicin treated samples, while vancomycin samples showed a slightly reduced survival of persisting cells on the BHIS agar containing sodium taurocholate. Taurocholate is a germinant that will promote germination of any spores present.

A reduction in ATP-bioluminescence was observed with metronidazole concentrations ≥0.5 μg/mL (**Figure 1A**), in line with the MIC of 0.5 μg/mL (**Table 1**), and the concentration necessary (≥0.5 μg/mL) to observe no growth from 5 μL aliquots from a replicate assay plate spotted on BHIS agar (**Figure 1B**). Similarly, fidaxomicin at ≥0.03 μg/mL reduced ATP-bioluminescence (**Figure 1A**) at similar concentrations to the MIC of 0.016 μg/mL (**Table 1**) and to the levels (≥0.125 μg/mL) resulting in no growth on BHIS from 5 μL aliquots (**Figure 1B**). At supra-MIC concentrations of metronidazole and fidaxomicin, the ATP-bioluminescence assay readout was consistent with the baseline measurement.

For vancomycin, a trough in the luminescence at 4 μg/mL of antibiotic corresponded to reduced growth from spotted aliquots (4–8 μg/mL), with both comparing well to the traditional MBC determination (MBC = 8 μg/mL). However, as the concentration of vancomycin increased, more surviving bacteria were observed to grow and this mirrored an increase in luminescence (**Figures 1A,B**). As the concentration of vancomycin increased above 64 μg/mL the cells exhibited a second decline in luminescence resulting in a second trough at 256 μg/mL but luminescence again increased at concentrations of antibiotic greater than 256 μg/mL up to 2048 μg/mL (**Figure 1A**).

The mean ATP-bioluminescence assay plate *Z’* score was 0.70 ± 0.03 (measured for strain 630 with *n* = 22), which indicated the assay quality was excellent (Zhang et al., 1999). The assay had a low average background signal (mean RLU of media = 2.73 × 10^3^ ± 0.05) relative to average positive growth (no compound) control wells (mean RLU 1.82 × 10^5^ ± 0.03). This resulted in a good signal to noise ratio (mean 68-fold ± 3). Importantly, the wells exhibiting the paradoxical Eagle effect had luminescence signals above the limit of detection (baseline media RLU ± 3 SEM).

### Assessment of Eagle-Type Resistance in *C. difficile* to Other Glycopeptide Antibiotics

**FIGURE 2:**
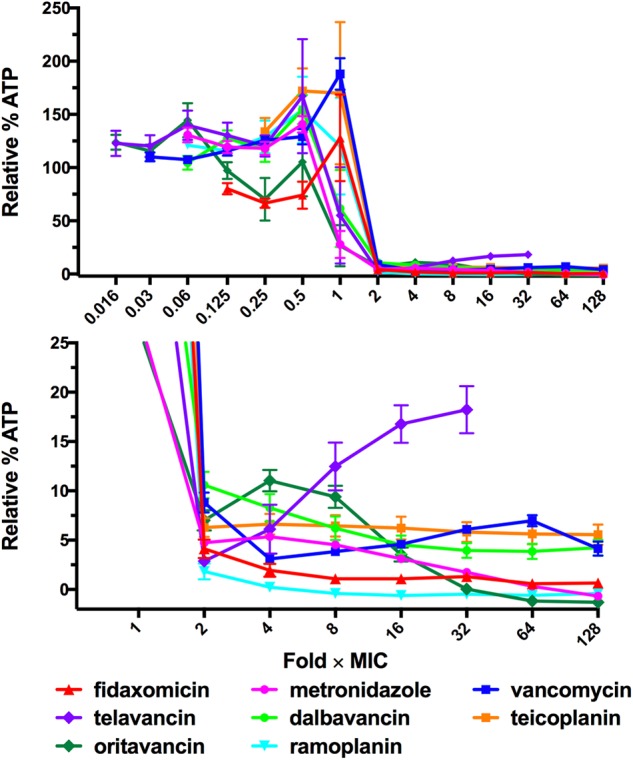
ATP-Bioluminescence after 24 h incubation of *C. difficile* 630 with the lipoglycopeptides telavancin, dalbavancin teicoplanin, and oritavancin and the lipoglycodepsipeptide ramoplanin. Results are normalized to fold × MIC. Minimum *n* = 6 ± SEM. Top: complete graph, bottom: expansion of top panel with % Relative Luminescence ≤25%.

Given that the Eagle effect was detected for vancomycin, one of the main therapeutic treatments of *C. difficile*, but not for metronidazole or fidaxomicin, we next examined other glycopeptide antibiotics for the Eagle effect in *C. difficile* strain 630. Telavancin, dalbavancin, teicoplanin, and oritavancin (lipoglycopeptides) and ramoplanin (lipoglycodepsipeptide) were compared to vancomycin (**Figure 2**). Telavancin also showed evidence of an Eagle effect, producing a marked increase in luminescence above a trough concentration of 0.25 μg/mL (2 × MIC). In contrast, dalbavancin treatment resulted in progressive reduction of luminescence from the MIC to about ∼4% relative luminescence at 16 × MIC (0.03–0.5 μg/mL). Further increases in dalbavancin concentration (32–128 × MIC) had no effect on the stabilized baseline. Teicoplanin at 2 × MIC and higher concentrations (128 × MIC) showed a consistent amount of relative luminescence at 4% relative luminescence ∼6%. Oritavancin displayed a similar profile to metronidazole with relative luminescence levels reduced in a concentration dependent manner from 4 × MIC down to a baseline level at 128 × MIC. Ramoplanin reduced relative luminescence from 2 × MIC to the baseline level up to 128 × MIC, consistent with the bactericidal response to fidaxomicin. Thus, vancomycin and telavancin both induced Eagle-type resistance, while teicoplanin and dalbavancin appeared to be bacteriostatic and oritavancin and ramoplanin appeared to be bactericidal.

### Eagle-Type Resistance to Vancomycin Was Conserved Between Different Pathogenic *C. difficile* Strains

**FIGURE 3:**
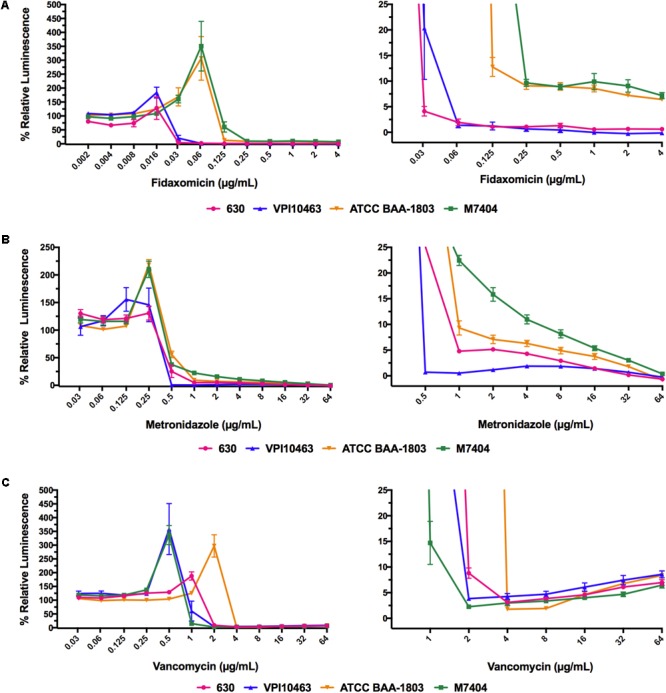
ATP-bioluminescence response of four strains of *C. difficile* was similar for each antibiotic. **(A)** fidaxomicin, **(B)** metronidazole, and **(C)** vancomycin. Left panels: complete graph, right panels: expansions of left panel with % Relative Luminescence ≤25%. Minimum *n* = 6 ± SEM.

The ATP-bioluminescence assay was next used to determine the profiles of two “hypervirulent” NAP01/027 strains (ATCC BAA-1803 and M7404) and a high-level toxin producer VPI10463 strain (ATCC 43255) when treated with metronidazole, vancomycin, and fidaxomicin (**Figure 3**). Overall, the ATP-bioluminescence profile in response to each antibiotic was consistent for all strains tested. Increasing concentrations of metronidazole steadily decreased luminescence for the 630 and NAP1/027 strains, while VPI10463 treated with all concentrations ≥0.5 μg/mL displayed overall low luminescence <2% (**Figure 3B**). Fidaxomicin had a similar trend to metronidazole for all strains, with increasing concentrations of antibiotic reducing luminescence (**Figure 3A**). Interestingly, fidaxomicin treatment of both NAP1/027 strains led to a higher relative luminescence (viability) compared to the 630/VPI10463 strains at concentrations of antibiotic higher than the MIC. Vancomycin showed the same trend observed for the 630 strain in the additional strains (**Figure 3C**), with an increase in luminescence above the trough point (typically 2–4 × MIC). Therefore vancomycin also exhibits the Eagle effect in these clinically relevant strains.

## Discussion

The Eagle effect is a paradoxical “more-drug-kills-less” mode of resistance to different classes of antibiotics, and is typically identified during MBC determinations by CFU-based plating methods. The Eagle effect is different from spontaneous resistance because bacteria persisting at higher concentrations of antibiotic have been shown to have the same MIC and paradoxical response in subsequent assays (Eagle, 1948). The traditional MBC assay has limited scalability, particularly when performed in an anaerobic chamber or anaerobic jars as required for anaerobic bacteria such as *C. difficile*. We therefore developed and assessed a higher-throughput ATP-bioluminescence based method to facilitate our investigations of the Eagle effect in *C. difficile*. Using ATP as a marker for viability, we have demonstrated that *C. difficile* can survive concentrations of vancomycin up to at least 2048 μg/mL *in vitro*. This result is consistent with those of Levett (1991), who reported the paradoxical survival of *C. difficile* at a concentration of vancomycin = 1000 μg/mL in contrast to the bactericidal concentration of 4 μg/mL. These high concentrations of vancomycin are therapeutically relevant, as the concentration achieved in the colon has been reported to be 520–2200 μg/mL (Young et al., 1985).

Vancomycin causes concomitant collateral damage to the resident gut microbiota *in vivo*, which is thought to contribute to treatment failure by disturbing the microbiome *in vivo* (Louie et al., 2012). High doses will likely cause more collateral damage and may not result in improved killing of *C. difficile* persister cells *in vivo* compared to lower doses, and may indeed prove less efficacious due to the Eagle effect. Nevertheless, increasing the dose of vancomycin is still a strategy used to treat severe CDI (Cohen et al., 2010). Recurrent CDI is complex, with multiple factors affecting cure rates and the Eagle effect is unlikely to account for all cases of vancomycin failure. However, the bactericidal nature of fidaxomicin may contribute to improved patient outcomes for this agent. Therefore, the clinical relevance of the Eagle effect for treating CDI with vancomycin should be more closely investigated, and it should be determined for potential new CDI antibiotics.

At high concentrations of antibiotic, the carryover of antibiotic to the agar plate used for traditional microbroth MBC determinations inhibits growth of persisting bacteria (Levett, 1991). In order to study Eagle-type resistance at concentrations relevant to that achieved in the colon, bacteria may need to be washed to remove excess antibiotic being carried over to agar plates. This procedure further increases the cumbersome nature of studying multiple concentrations of antibiotics. Alternatively, inclusion of additives in the agar, such as β-lactamases to inactive β-lactam antibiotics, or activated charcoal to sequester antimicrobial agents, can also prevent antibiotic carryover effects. An advantage of the ATP-bioluminescence detection method described here was that no washing steps or additives were required because detection of persistent bacteria did not depend on bacteria growing into colonies. Therefore, the cell viability response to vancomycin could be examined over a wider concentration range (**Figure 1A**).

We examined a set of glycopeptide-based antimicrobials in order to determine whether structural differences impacted on Eagle-type resistance. Telavancin, dalbavancin, and oritavancin all contain lipophilic elements that can insert into the bacterial membrane, in addition to other structural variations including variations in sugar substituents and alterations to the core peptide backbone. These changes result in differences in the dimerization properties and binding to the peptidic target on lipid II, potency, and activity against vancomycin resistant bacteria (Butler et al., 2014; Cheng et al., 2014b). *C. difficile* also displayed Eagle-type resistance to telavancin, which retains the closest core structure to vancomycin. Dalbavancin and teicoplanin, which both possess an additional biaryl ether cyclic backbone, appeared bacteriostatic. Oritavancin, also with a vancomycin-type core but containing an additional sugar substituent, was the only lipoglycopeptide examined to result in a concentration dependent decrease in ATP to baseline levels with increasing antibiotic concentrations. Oritavancin did still exhibit a subtle indication of an Eagle effect as there was less luminescence at 2 × MIC compared to 4 × MIC. Ramoplanin, a glycodepsipeptide that also inhibits cell wall synthesis, reduced luminescence to baseline levels at 2–4 × MIC. The observed bactericidal activity of oritavancin and ramoplanin at concentrations higher than the MIC is consistent with previous studies against Gram-positive organisms (Johnson et al., 1992). *Enterococcus faecalis* has been shown to be killed in a concentration dependent manner by oritavancin but with paradoxical killing by vancomycin (McKay et al., 2009). Notably, oritavancin and ramoplanin, which can overcome the Eagle effect, have been shown to cause membrane depolarization (Belley et al., 2010; Cheng et al., 2014a) in addition to bacterial ligand binding. Therefore, antibiotics operating through multiple modes of action appear to be effective at preventing the paradoxical Eagle effect in *C. difficile*.

The four representative *C. difficile* strains examined for potential Eagle effects induced by vancomycin, metronidazole, or fidaxomicin were selected on the basis of different ribotypes, toxinotypes, clades, and previous studies on these *C. difficile* strains, including animal models of antibiotic efficacy. All four strains reproducibly displayed the Eagle-type resistance to vancomycin (up to the maximum 64 μg/mL concentration tested), but not to metronidazole or fidaxomicin. This result highlights that the Eagle effect is not an isolated phenomenon in pathogenic *C. difficile* strains and indeed, may be quite prevalent. The percentage of strains of the same species displaying Eagle-type resistance is known to vary. For example, 5 of 7 strains of *Streptococcus faecalis* and 3 of 7 strains of *S. aureus* had Eagle-type resistance to penicillin in one of the initial reports of the Eagle effect (Eagle, 1948). The ATP-bioluminescence assay described here provides a facile procedure that enables future investigation of additional *C. difficile* strains to establish a better understanding of the prevalence of Eagle-type resistance in multiple strains, and for different antimicrobial agents. This could provide a clearer picture of the potential impact of the Eagle effect in *C. difficile* in a clinical setting. Understanding why some glycopeptides induce the Eagle effect and others do not, could also give insight into the molecular mechanism of Eagle-type resistance to vancomycin in *C. difficile* and contribute to our broader knowledge of antimicrobial resistance mechanisms in bacteria.

## Conclusion

A commercial ATP-bioluminescence assay was found to be a more convenient alternative to the current gold standard CFU-based methods to assess the Eagle effect in *C. difficile*. This assay was used to detect the Eagle effect in *C. difficile* at supra-MIC, clinically relevant levels of vancomycin. When determining the MBC of vancomycin and telavancin against pathogenic *C. difficile* strains, a wide range of antibiotic concentrations that cover clinically relevant concentrations should be plated to probe for potential paradoxical effects. The Eagle effect with *C. difficile* does not appear to be target specific, as other glycopeptide antibiotics that also inhibit peptidoglycan synthesis: teicoplanin, dalbavancin, and oritavancin, did not exhibit the effect. The Eagle effect should be examined in a wider variety of *C. difficile* strains as Eagle-type resistance to vancomycin may have clinical implications. We recommend that the MBC be examined at concentrations well above the MIC for new drugs under development for treatment of CDIs in order to detect potential Eagle-type resistance. If an Eagle effect is detected in the traditional MBC assay, the ATP-bioluminescence method could be used to investigate this effect in different bacterial strains and for analogs of the drug candidate with greater throughput. The ATP-bioluminescence assay thus facilitates rapid investigation of the Eagle effect and complements the traditional MBC approach, which can be employed to validate results from the ATP-bioluminescence assay.

## Author Contributions

AJ designed the study, carried out the experiments, analyzed the data, and wrote the manuscript. KH synthesized the dalbavancin used in this study. AP carried out the experiments. TK, MB, and MC analyzed the data and wrote the manuscript.

## Conflict of Interest Statement

MB and MC are inventors on patent application WO 2015/117196-A1, which describes novel glycopeptides potentially suitable for treating *C. difficile* infections. The remaining authors declare that the research was conducted in the absence of any commercial or financial relationships that could be construed as a potential conflict of interest. The reviewer SS and handling Editor declared their shared affiliation.

## Footnotes

1 https://www1.actelion.com/investors/news-archive?newsId=2111437
